# Factores para herpes simple: Revisión de literatura

**DOI:** 10.21142/2523-2754-1001-2022-099

**Published:** 2022-03-30

**Authors:** Lisette Begazo, Alysson Morante, Carlos Espinoza Montes

**Affiliations:** 1 Carrera de Estomatología, Facultad de Ciencias de la Vida y Salud. Universidad Científica del Sur, Lima, Perú. 100062307@cientifica.edu.pe, 100063806@ucientifica.edu.pe, cespinozam@cientifica.edu.pe Universidad Científica del Sur Carrera de Estomatología Facultad de Ciencias de la Vida y Salud Universidad Científica del Sur Lima Peru 100062307@cientifica.edu.pe 100063806@ucientifica.edu.pe cespinozam@cientifica.edu.pe

**Keywords:** herpes simple, inmunosupresión, latencia del virus, herpes simplex, immunosuppression, virus latency

## Abstract

El virus del herpes simple, después de la primera infección, puede permanecer latente en los ganglios y reactivarse en cualquier momento debido a ciertos factores o estímulos, lo que ocasiona efectos en el paciente. El objetivo de esta revisión de literatura es actualizar la información científica sobre los factores que predisponen la reactivación del virus del herpes simple. Se realizó una revisión bibliográfica exhaustiva en las bases de datos LILACS, SciELO, PubMed y Scopus, mediante las palabras clave “herpes simple”, “reactivación”, “latencia” y “riesgos”. Entre los factores descritos están la reactivación por inmunosupresión debido a la ingesta de inmunosupresores o la terapia oncológica. Otro factor es el estrés psicológico, en el cual las hormonas liberadas actúan sobre señales mediadoras de la reactivación, así como la exposición solar, que reactiva al virus en las neuronas infectadas, y la infección en mujeres gestantes, que puede desencadenar complicaciones en el feto y en el momento del parto.

## Abstract (en)

Herpes simplex virus, after the first infection, can remain dormant in the lymph nodes and reactivate at any time due to certain factors and/or stimuli causing effects in the patient. The purpose of this literature review is to update scientific information on the factors that predispose to the reactivation of herpes simplex virus. A comprehensive literature review was conducted in the LILACS, SciELO, PubMed, Scopus and the following keywords were used: herpes simplex, reactivation, latency and risks. A factor is reactivation by immunosuppression due to the intake of immunosuppressants or oncological therapy. Another factor is psychological stress, in which the hormones released act on mediating signs of reactivation. Sun exposure that reactivates the virus in infected neurons and infection in pregnant women can trigger complications in the fetus and delivery.

## INTRODUCCIÓN

Según información brindada por la Organización Mundial de la Salud (OMS), la infección por el virus del herpes simple puede ser consecuencia del virus herpes simple de tipo 1 (VHS-1) o del virus herpes simple de tipo 2 (VHS-2). A pesar de que la infección por virus herpes simple es muy común en la cavidad oral, se tiene escasa información referente al mecanismo de esta infección y su traslado al ganglio trigémino, debido a que aún no se tienen modelos de estudio que esclarezcan cómo se da esta migración ^1^. Existe evidencia científica de que el VHS-1 causa mayormente infecciones primarias, mientras que las infecciones por VHS-2 son, en su mayoría, por recurrencia y causan herpes genital. A pesar de ello, el VHS-1 también puede causar herpes genital. Magdaleno *et al*. observaron que, en España, hubo una mayor prevalencia de herpes genital causado por el VHS-1 en mujeres alrededor de los 26 años ^2^. Por otro lado, Durukan *et al*. observaron que, en Australia, el VHS-1 ha causado herpes anogenital en mayor proporción que el VHS-2, sobre todo en pacientes jóvenes mujeres y en hombres que tienen relaciones sexuales con hombres ^3^.

Debido a la infección por VHS, se pueden dar diversas complicaciones como encefalitis, la cual genera alteraciones en el comportamiento, convulsiones y un detrimento de la memoria. Se demostró que la terapia con aciclovir no brindó una mejora en las pruebas neuropsicológicas posteriores a 12 meses ^4^. La queratitis es una de las principales causas de ceguera en el mundo y uno de sus factores causales es la infección por VHS-1; este virus induce una respuesta inflamatoria crónica que afecta la cicatrización y produce neovascularización y adelgazamiento de la córnea ^5^. El herpes neonatal, a pesar de no ser muy frecuente, puede causar riesgos graves al neonato y generar una mortalidad y una morbilidad significativas. Por este motivo, la prevención, detección y terapia antiviral temprana son las principales medidas que deben tomarse. Se recomienda a las madres gestantes realizarse una cesárea, en caso presenten lesiones herpéticas genitales para evitar así el contacto del recién nacido con el virus ^6^^,^^7^.

El ADN (ácido desoxirribonucleico) latente del virus herpes simple detecta estímulos que llevan a su reactivación, como la exposición a la luz solar debido a diversos efectos de la radiación UV en las neuronas, el estrés psicológico debido a la acción de hormonas liberadas sobre señales comunes mediadoras, la supresión de la respuesta inmunitaria debido a la ingesta de inmunosupresores o terapia oncológica y las complicaciones en el feto, como la infección en la gestación y en el momento del parto, e incluso la muerte fetal. Todos estos factores pueden combinarse y están asociados con la reactivación del virus ^8^. 

El objetivo de esta actualización de la literatura es identificar los factores de riesgo para la infección y la reactivación del virus herpes simple, teniendo en cuenta los factores intrínsecos y extrínsecos que contribuyen a su reactivación.

## MATERIALES Y MÉTODOS

Se han utilizado las principales fuentes de búsqueda de literatura como LILACS, SciELO, PubMed y Scopus, mediante las siguientes palabras clave: “herpes simple”, “reactivación”, “latencia” y “riesgos”. Se utilizó el término “herpes virus” para la selección de artículos y se revisaron 43 artículos, los cuales fueron incluidos en esta revisión. Como criterios de inclusión se consideró la literatura desde el 2015 hasta la actualidad, los estudios descriptivos y transversales retrospectivos, y los artículos en español, inglés y portugués. Los criterios de exclusión fueron las cartas al editor, las opiniones de expertos y los reportes de casos.

### Virus del herpes simple

El virus del herpes pertenece a la familia *Herpesviridae*, que incluye 8 tipos subdivididos en 3 grupos: alfa, beta y gamma. En el grupo alfa, también conocido como la subfamilia *Alphaherpesvirinae*, se encuentran el VHS-1, el VHS-2 y el virus de la varicela zóster. El virus herpes simple es un virus ADN con envoltura, su infección comienza con lesiones en piel o mucosa, y la replicación en las células epiteliales; posteriormente, en los nervios autónomos y, finalmente, se aloja en los ganglios neuronales, donde permanece latente. El VHS-1 se adquiere en la infancia y es responsable de las lesiones orofaciales y de las lesiones vesiculares en la recurrencia sintomática. El VHS-2, a diferencia del VHS-1, se adquiere al inicio de la vida sexual y presenta principalmente úlceras genitales. Sin embargo, ambos virus pueden tener las mismas manifestaciones clínicas ^9^^,^^10^.

### Infección en mujeres gestantes

El virus del herpes simple en mujeres gestantes es poco frecuente; sin embargo, supone un riesgo mayor ya que se puede transmitir al bebé ^7^. La infección del VHS durante la gestación aumenta las probabilidades de tener un parto prematuro, aborto espontáneo y muerte fetal ^11^. En un estudio realizado en un hospital de Paraguay, se observó que el VHS afectó al 32,5% de las mujeres embarazadas -con mayor prevalencia entre los 25 y 39 años-, las cuales eran solteras y provenían de zona urbana ^12^. Aunque es poco frecuente, el VHS-1 y VHS-2 pueden encontrarse tanto en la placenta como en la sangre del cordón umbilical e infectar al feto, lo cual puede causarle daños graves al recién nacido ^13^^,^^14^. Por ello, es importante tener todos los controles y medidas de prevención durante el embarazo. El VHS-2 también afecta a las gestantes, sobre todo a las que tienen antecedentes de ETS ^15^, ya que puede seroconvertirse durante el embarazo por factores de riesgo como la poligamia, tener múltiples parejas sexuales o ser portador de VIH ^16^. Los bebés de las mujeres que padecen herpes genital sintomático pueden presentar lesiones en la piel al momento del nacimiento en caso no se tomen las precauciones para evitar herpes neonatal ^17^. La infección del virus del herpes simple durante el embarazo puede causar hepatitis aguda, aunque esto no es muy frecuente, pero cuando se presenta puede poner en peligro la vida del paciente ^18^.

### Reactivación por exposición a la luz solar

La fotosensibilidad puede presentarse en hombres y mujeres de diferentes grupos étnicos; los factores ambientales y genéticos pueden relacionarse con su aparición. Esta fotosensibilidad se da por una reacción anormal entre un compuesto reactivo de la piel y un componente del espectro electromagnético de la luz solar, la cual tiene subdivisiones como la radiación ultravioleta A (esta suele ser la más común y la más asociada a las fotodermatosis), la B y la radiación gamma ^19^. Los rayos x utilizados en la radioterapia también pueden activar al VHS-1 y al VHS-2; sin embargo, es necesario esclarecer cómo su reactivación afecta a esta terapéutica ^20^. Las fotodermatosis se dan por una interacción irregular de la luz UV y la piel, y la mayor parte son reguladas mediante el sistema inmunológico. Asimismo, las dermatosis fotoagravadas son aquellas afecciones cutáneas preexistentes que empeoran debido a esta exposición ^19^.

En un estudio, la tasa de recurrencia del herpes labial puede disminuir con el uso de bloqueador solar debido a que el 50 % de los voluntarios que no lo utilizó presentó herpes labial recurrente por exposición a la luz solar ^21^; no obstante, se requiere de más estudios *in vivo* para comprender y prevenir la recurrencia del herpes labial ante factores ambientales. Por otro lado, existe evidencia que indica que el uso de protector solar no es una medida preventiva para la recurrencia del VHS ^22^^,^^23^. Actualmente, se cuenta con poca información acerca del efecto de la radiación ultravioleta en el microbioma de la piel y cómo la respuesta inmune se ve afectada. Además, existe una escasa evidencia clínica que indique la relación entre la radiación UV y la recurrencia del VHS ^24^.

### Reactivación por estrés psicológico

Se conoce que bajo situaciones de estrés ocurre daño oxidativo, lo que genera un aumento de glucocorticoides y una disminución de las hormonas tiroideas que crea un desequilibrio en el balance del organismo; esto tiene como resultado la replicación viral del VHS-1 ^25^. Las hormonas liberadas por el estrés como la epinefrina y la corticosterona producen efectos sobre el VHS-1, y la replicación del ADN del VHS-2 en las neuronas simpáticas. Además, el VHS-2 ingresa y se aloja en los axones terminales de las neuronas sensoriales; sin embargo, también puede recurrir en los ganglios autónomos ^26^^,^^27^.

Los factores estresantes podrían activar proteínas que actúan como señales comunes mediadoras de pasos importantes para la reactivación de la latencia como la c-Jun quinasa N-terminal y el receptor de glucocorticoides; además, el estrés actúa en otros miembros de la subfamilia *Alphaherpesvirinae*^28^.

Se debe tener en cuenta también que existen factores neuronales que inhabilitan la apoptosis, lo cual facilita la supervivencia de las células infectadas ^25^. Las evidencias indican que el VHS-2 tuvo mayor prevalencia en mujeres con un nivel de angustia elevado, estado de ánimo lábil y un endeble control emocional, e indica que la variación en la estabilidad emocional, la regulación de las emociones y la angustia psicológica se relacionan con la recurrencia del VHS-2. Concluyeron que el sistema inmunológico se ve suprimido por la angustia psicológica, la cual sería causante de la diseminación viral del VHS-2 ^29^. Estudios epidemiológicos y experimentales evidencian la presencia de una relación entre los procesos neurodegenerativos típicos de la enfermedad de Alzheimer y la recurrencia del VHS-1. Sin embargo, se necesitan más estudios para corroborar este vínculo ^30^.

### Reactivación por inmunosupresión

Cuando la respuesta inmunitaria de las personas se ve deteriorada, la infección por el VHS es más viable, por lo que se entiende que el virus afecta más a los pacientes inmunosuprimidos ^20^. Por ello, uno de los factores predisponentes para el virus del herpes simple es el uso de fármacos inmunosupresores, por ejemplo, en pacientes con trasplante de riñón, ya que pueden generar lesiones bucales ^31^. Similarmente, pacientes con terapia oncoespecífica se encuentran más expuestos a la reactivación del virus del herpes simple porque tienen su sistema inmunológico debilitado por el tratamiento ^32^. Asimismo, la tiroiditis autoinmune de Hashimoto puede ser un factor predisponente para la infección del virus del herpes, específicamente para el herpes virus humano 6A ^33^.

Otra de las enfermedades que afectan el sistema inmunológico y suponen un riesgo mayor para la infección de VHS es el lupus eritematoso sistémico, ya que requiere un tratamiento inmunosupresor. En un estudio, se observó que la prevalencia del VHS en pacientes adultos con lupus eritematoso sistémico fue alta ^34^. Algunos de los factores de riesgo asociados pueden ser la edad, la exposición al VHS y el consumo de esteroides ^35^.

La esofagitis herpética afecta a los pacientes inmunosuprimidos y se presenta como úlceras en el esófago, lo cual se puede observar mediante una endoscopía ^36^. Aunque comúnmente la esofagitis es causada por el VHS-1, también la puede causar el VHS-2 en pacientes inmunocompetentes, aunque en menor proporción ^37^.

## DISCUSIÓN

Luego de la infección primaria, el virus del herpes simple permanece en los ganglios en un estado de latencia, ya que no se expresa el genoma viral; sin embargo, puede haber periodos de reactivación debido a algunos factores predisponentes tales como la infección en gestantes, la reactivación por luz solar, el estrés psicológico y la reactivación por inmunosupresión. Esta infección produce lesiones orales, por ejemplo, ampollas en los labios. Aunque la mayoría de los pacientes en la primera infección no presentan signos, existen casos en los que muestran gingivoestomatitis herpética. El VHS-2 causa lesiones genitales; sin embargo, no siempre es así, ya que debido a las prácticas sexuales ambos virus pueden causar las mismas características clínicas ^2^^,^^38^.

Existe evidencia que indica que el virus del herpes simple también afecta a las gestantes y esto pone en riesgo la salud del hijo. En las embarazadas, se observó mayor prevalencia en las mujeres jóvenes, esto podría deberse a tener múltiples parejas sexuales o ser portador de VIH, ya que esto aumenta el riesgo de seroconversión de virus del herpes simple tipo 2 ^(12, 16)^. Similarmente, Patton *et al*. refieren que las embarazadas con múltiples parejas sexuales tienen un mayor riesgo de contraer la infección primaria tanto del VHS-1 como del VHS-2, lo cual supone un mayor riesgo de infectar al recién nacido ^34^. Pichler *et al.* describieron un caso de transmisión transplacentaria del VHS-1 en gemelos, los cuales tuvieron un nacimiento prematuro y presentaron múltiples lesiones cutáneas; también se vieron afectadas la mucosa de los ojos y genitales ^39^. Por otro lado, Sivarajah *et al*. observaron que las mujeres con VIH positivo que contraen el VHS-2 durante el embarazo corren un mayor riesgo de trasmitirle el VIH a sus hijos ^40^.

Un estudio reporta que el protector labial en barra evita la recurrencia de herpes labial ante la exposición solar en condiciones naturales; al grupo experimental se le aplicó una crema con un factor de protección solar de 30, la cual generó una menor recurrencia del herpes comparado con el grupo control ^21^. La formulación de una nueva crema labial con filtro UV como agente protector ante los factores ambientales que desencadenan la recurrencia del herpes labial generó resultados positivos en la protección ante la radiación UV, sequedad y agrietamiento ^23^. Por otro lado, Chi *et al*. refieren que el efecto preventivo del protector solar es incierto, pues este resultó efectivo para evitar la recurrencia de herpes labial frente a la exposición a luz ultravioleta experimental; sin embargo, no fue así frente a la luz solar ^22^.

Después de producirse la infección primaria, el VHS-1 se mantiene latente y se reactiva por estrés al alterarse el equilibrio inmunológico que afecta las defensas del huésped. Debido al estrés, se genera un desequilibrio de glucocorticoides y hormonas tiroideas, lo cual da como resultado la replicación del VHS-1^25^. Del mismo modo, el aumento de hormonas como la epinefrina y la corticosterona también tiene efectos en la replicación del VHS-2, sobre todo en las neuronas autónomas, debido a las variaciones hormonales producidas por el estrés; esto demuestra el importante rol del sistema autónomo sobre la patogénesis del VHS ^26^. Igualmente, las proteínas involucradas en la replicación del VHS latente participan en este proceso por inducción de factores estresantes ^28^.

Según las evidencias encontradas la inmunosupresión, también es un factor de riesgo para la reactivación del virus del herpes simple. Estrada *et al*. observaron que en los pacientes con trasplante de riñón hubo una mayor prevalencia de reactivación del herpes simple en los hombres mayores de 60 años y que tomaban medicamentos inmunosupresores, en los cuales se observaron lesiones bucales ^31^. Similarmente, en otro estudio de Estrada *et al*. en pacientes con terapia oncoespecífica se observó la mayor recurrencia del virus del herpes simple en el mismo grupo de edad y sexo, pero esta vez en pacientes que recibían quimioterapia; ellos también presentaron lesiones bucales. En ambos estudios, las alteraciones celulares más relevantes fueron la célula gigante multinucleada y la necrosis de células infectadas ^32^. Por otro lado, en pacientes con lupus eritematoso, se observó que la reactivación del virus del herpes simple tiene mayor prevalencia en pacientes mayores de 18 años y que otro factor de riesgo en estos pacientes es el consumo diario de esteroides como la prednisolona ^35^.

Esta revisión se realizó de acuerdo con el criterio del investigador y se determinaron otros factores predisponentes; sin embargo, se encontró muy escasa evidencia acerca de la asociación con la menstruación, la fiebre y la reactivación del virus del herpes simple en mujeres, por lo cual no se incluyeron en la presente revisión para su análisis ^36^^,^^37^.

## CONCLUSIÓN

El virus herpes simple, al alojarse en los ganglios del sistema nervioso, se ve expuesto a diversos estímulos que llevan al virus a recurrir. Entre los factores intrínsecos que predisponen la reactivación del VHS se mencionan la inmunosupresión, el estrés psicológico y la infección en gestantes, mientras que la luz solar vendría a ser un factor extrínseco. Estos factores pueden darse paralelamente y propiciar la reactivación del virus en estado latente. En el caso de la infección en el embarazo, es más frecuente en mujeres jóvenes y que tienen múltiples parejas sexuales. Se requiere mayor evidencia clínica acerca de la relación entre los rayos ultravioleta y la reactivación del VHS. El estrés también es un factor en la reactivación del virus, pues tiene efecto sobre las neuronas simpáticas y autónomas infectadas. Por otro lado, aquellos pacientes que se encuentran con el sistema inmunológico debilitado también están más expuestos a la reactivación del VHS.

## Fuente de financiamiento:

Autofinanciado
